# Activation of Akt characterizes estrogen receptor positive human breast cancers which respond to anthracyclines

**DOI:** 10.18632/oncotarget.17167

**Published:** 2017-04-17

**Authors:** Synnøve Yndestad, Eilin Austreid, Ida R. Svanberg, Stian Knappskog, Per E. Lønning, Hans P. Eikesdal

**Affiliations:** ^1^ Section of Oncology, Department of Clinical Science, University of Bergen, Bergen, Norway; ^2^ Department of Oncology, Haukeland University Hospital, Bergen, Norway

**Keywords:** Akt, doxorubicin, drug resistance, estrogen receptor, breast cancer

## Abstract

Anthracyclines are key components of human breast cancer chemotherapy. Here, we explored the role of Akt signaling in anthracycline resistance.

The antitumor activity of doxorubicin and Akt inhibitor A-443654 alone or combined was examined in estrogen receptor (ER) positive and negative human breast cancer cell lines. Further, we examined mRNA changes induced by anthracyclines in locally advanced breast cancers biopsied before and after treatment in two clinical trials.

Doxorubicin increased Akt phosphorylation in ER positive MCF7 and T47D cell lines, with no effect in ER negative MDA-MB231 breast cancer cells. A-443654 was significantly more cytotoxic in doxorubicin-resistant compared to doxorubicin-naïve MCF7. This difference was not observed in MDA-MB231. Among 24 patients, *AKT1* gene expression increased 24 hrs after the initial epirubicin exposure in ER positive tumors responding to therapy (n=6), as compared to ER positive non-responders (n=7) or ER negative tumors (n=11). In contrast, *AKT1* mRNA changes after 16 weeks of doxorubicin were unrelated to clinical response and ER status (n=30).

In conclusion, rapid Akt activation was observed in ER positive breast cancers which responded to anthracyclines. Increased cytotoxicity of A-443654 in doxorubicin-resistant MCF7 cells indicates a possible role for Akt inhibitors in ER positive breast cancers where chemoresistance evolves.

## INTRODUCTION

Phosphatidylinositol-4,5-bisphosphate 3-kinase (PI3K)-Akt-mammalian target of rapamycin (mTOR)-S6 kinase (S6K) signaling (in short: PI3K signaling) is upregulated in 25% of human breast cancers and has been associated with resistance to endocrine as well as HER2 directed therapy [1–3]. *PIK3CA*, encoding the p110α subunit of PI3K, harbors activating mutations in up to 45% of luminal A breast cancers [4], which are typically estrogen receptor (ER) positive tumors. Thus, therapeutic inhibition of the PI3K signaling pathway with the mTOR inhibitor everolimus can be used to counteract acquired resistance to aromatase inhibitors and prolong survival among patients with ER positive breast cancer [3]. Moreover, activating *PIK3CA* mutations are observed in ER negative breast cancer as well [4], and mTOR inhibition, combined with trastuzumab and paclitaxel, prolonged progression-free survival significantly among patients with hormone receptor negative, HER2 positive breast cancer [5]. However, the potential to treat chemoresistant breast cancer by inhibiting PI3K signaling has not been thoroughly addressed thus far.

Phosphatase and tensin homolog (PTEN) is the main endogenous inhibitor of PI3K activation [6]. While experimental studies revealed loss of PTEN function to be associated with reduced sensitivity to doxorubicin in breast and prostate cancer models, chemosensitivity was restored by concomitant mTOR inhibition [7, 8]. Furthermore, increased Akt phosphorylation is observed in doxorubicin-resistant ER positive, but not in ER negative breast cancer cell lines [9–11]. In line with this, inhibitors of the PI3K-Akt-mTOR pathway can be employed to enhance anthracycline sensitivity in ER positive breast cancers [10, 11] Whereas the introduction of Akt inhibitors in clinical trials has been slower than PI3K and mTOR inhibitors [12], the key position of Akt as a signal hub for important pro-tumorigenic pathways [6] makes such trials highly relevant.

In the present work we assessed the influence of doxorubicin treatment on PTEN and Akt-mTOR-S6K signaling, and the interaction between doxorubicin and the Akt inhibitor A-443654 in ER positive and negative human breast cancer cell lines *in vitro* and *in vivo*. In particular, cell lines made resistant to doxorubicin by continous drug exposure were compared with doxorubicin-naïve cells to decipher the role of Akt-mTOR-S6K signaling in breast cancer chemoresistance. Furthermore, the short-term and long-term changes in *PTEN* and *AKT1* gene expression subsequent to anthracycline exposure were assessed in patients with locally advanced breast cancers.

## RESULTS

### Influence of doxorubicin treatment on Akt activity and PI3K signaling in doxorubicin-naïve breast cancer cell lines

A sublethal concentration of doxorubicin (24 hrs exposure) was established by the WST-1 assay, to facilitate subsequent assessment of increased cytotoxicity when the A-443654 Akt inhibitor was introduced. The IC30 was approximately 1.5-2.0 μM for MB231 and MCF7 and 0.5-1.0 μM for T47D (Supplementary Figure 1A). Based on this, doxorubicin was used at a concentration of 1.5 μM for MB231, 2 μM for MCF7 and 0.7 μM for T47D for the *in vitro* experiments. Each experimental setup was conducted in three parallel cell cultures.

**Figure 1:**
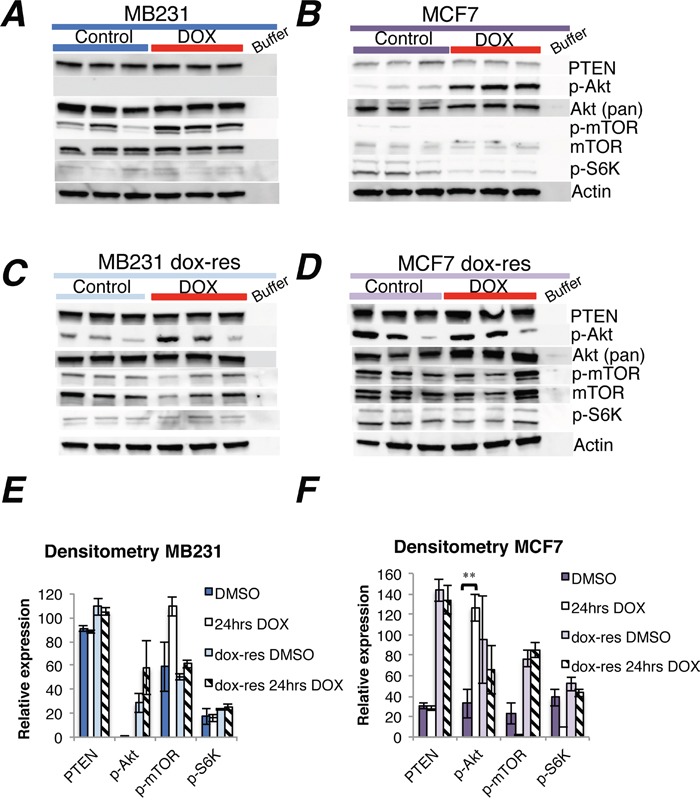
Doxorubicin treatment of doxorubicin-naïve and doxorubicin-resistant human breast cancer cell lines **(A-B)** Western blots of PTEN and Akt-mTOR-S6K signaling in MB231 and MCF7 breast cancer cells *in vitro*, either doxorubicin-naïve **(A-B)** or doxorubicin-resistant (**C-D**, dox-res). Drug exposure lasted 24 hrs, at either 1.5 μM for MB231 and 2 μM for MCF7 or an equivalent volume of DMSO (stock solvent for doxorubicin) for control wells, three independent experiments per group. Whole cell lysate, 30 μg protein loaded per lane. **(E-F)** Densitometries for western blots **(A-D)** depict the relative protein expression, normalized to actin. Phosphorylated Akt (p-Akt) and mTOR (p-mTOR) were normalized to actin and thereafter to total Akt and mTOR, respectively. Bars represent the mean protein expression for experiments performed in triplicate ± SEM. **p<0.01

Doxorubicin increased phosphorylated Akt (p-Akt) in the ER positive MCF7 and T47D human breast cancer cell lines (Figure 1B, Supplementary Figure 1B). In contrast, p-Akt was not influenced by doxorubicin in the ER negative MB231 cell line (Figure 1A). Whereas doxorubicin had no impact on PTEN protein levels in neither cell line, mTOR phosphorylation levels increased in MB231 and decreased in MCF7 cells (Figure 1A-1B), although not significant by densitometry (Figure 1E-1F). Phosphorylated S6K was weakly expressed in both cell lines, and a non-significant decrease in S6K phosphorylation levels was observed in the MCF7 cell line after doxorubicin exposure (Figure 1A-1B).

*AKT1* mRNA levels as determined by qPCR analysis remained unaltered 24 hours after doxorubicin exposure in all three cell lines (Supplementary Figure 2A). While *PTEN* mRNA levels decreased in MB231 cells, no change was observed in MCF7 and T47D cells subsequent to doxorubicin treatment (Supplementary Figure 2A). The reason why decreased *PTEN* mRNA levels did not translate into decreased PTEN protein levels in MB231 cells exposed to doxorubicin remains to be elucidated, but the rapid changes in gene expression induced by the chemotherapy could take longer to translate into a change in protein levels, due to a half-life of more than 8 hrs for PTEN [13]. Furthermore, there is no strong correlation between *PTEN* mRNA and PTEN protein levels in human breast cancer, which could be explained by post-transcriptional and post-translational mechanisms modifying protein expression and stability [14].

### Influence of doxorubicin treatment on Akt activity and PI3K signaling in doxorubicin-resistant cell lines

We performed the same experiments as outlined above in MB231 and MCF7 cells made resistant to doxorubicin through long-term doxorubicin exposure (see Methods & materials).

While doxorubicin exposure for 24 hours increased *AKT1* gene expression in doxorubicin-resistant MCF7 cells, no significant change in *AKT1* expression was observed subsequent to doxorubicin expression in MB231 cells (Supplementary Figure 2B). Notably, *PTEN* gene expression was profoundly reduced 24 hrs after doxorubicin exposure in doxorubicin-resistant MB231, whereas a minor *PTEN* increase was observed in doxorubicin-resistant MCF7 cells (Supplementary Figure 2B). While p-Akt increased at the protein level in doxorubicin-resistant compared to doxorubicin-naïve MB231 cells, no change in downstream signaling was observed. The level of p-Akt was increased in doxorubicin-resistant MB231 cells, compared to doxorubicin-naïve cells, but without any changes in downstream signaling (Figure 1A, 1C). In doxorubicin-resistant MCF7 cells, the protein levels of PTEN, p-Akt, mTOR and p-mTOR were higher compared to doxorubicin-naïve cells (Figure 1B, 1D). However, an additional pulse of doxorubicin treatment did not change PTEN or Akt-mTOR-S6K protein levels further in doxorubicin-resistant MB231 or MCF7 cells, compared to sham treatment (Figure 1C-D).

### Akt inhibition in doxorubicin-naïve and resistant MB231 and MCF7 cell lines

Next, we examined the cytotoxicity of the Akt inhibitor A-443654, alone or combined with doxorubicin, in the ER negative MB231 and ER positive MCF7 cell lines. Moreover, based on the increased Akt phosphorylation levels observed in the doxorubicin-resistant cell lines, we compared the doxorubicin-naïve and resistant cell lines with respect to Akt inhibitor cytotoxicity.

**Figure 2:**
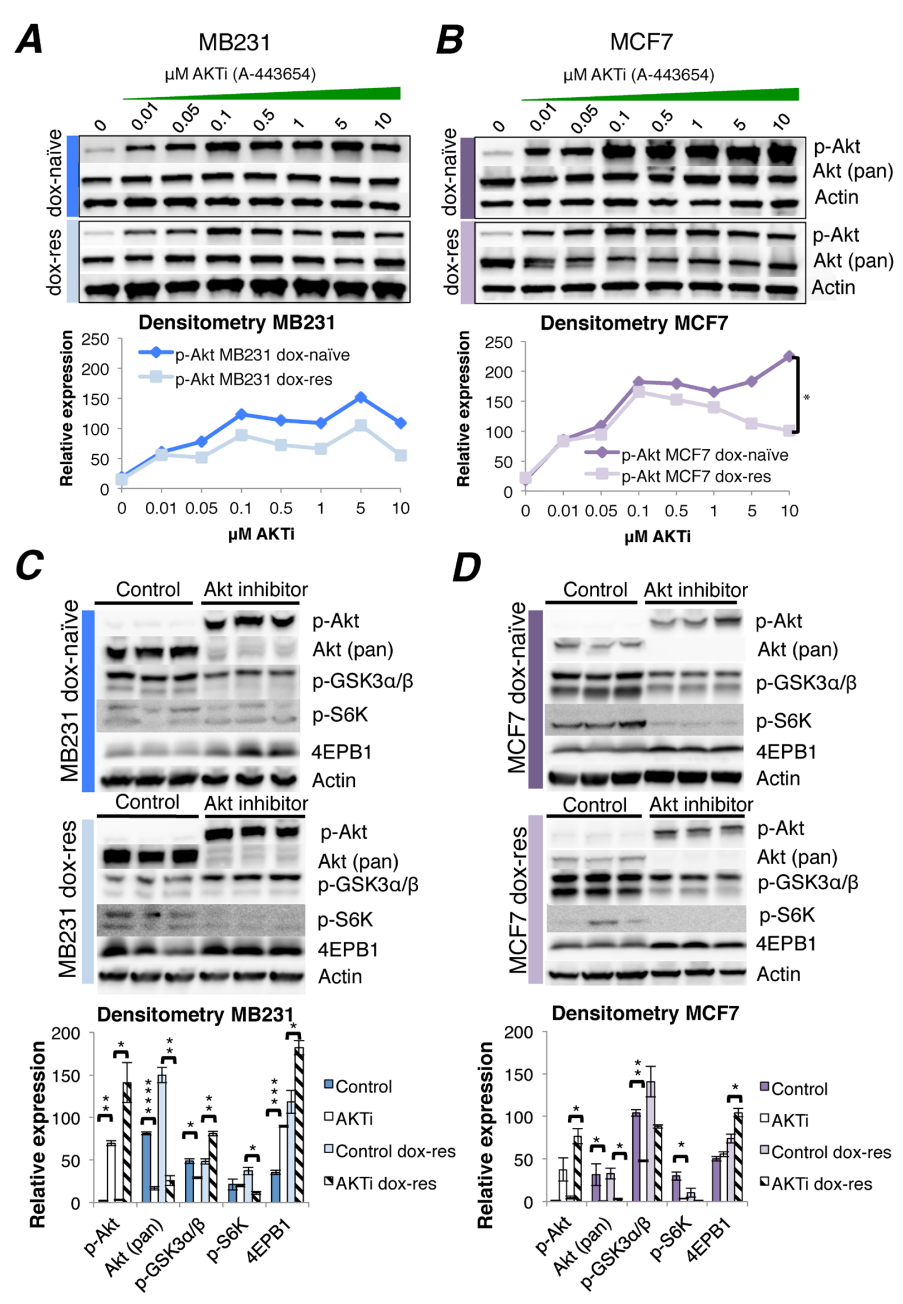
Akt inhibitor treatment of doxorubicin-naïve and doxorubicin-resistant human breast cancer cell lines **(A-B)** Western blots of Akt phosphorylation induced by increasing doses of the Akt inhibitor A-443654, 0-10 μM, 2 hrs exposure in doxorubicin-naïve or doxorubicin-resistant MB231 **(A)** and MCF7 **(B)** human breast cancer cells *in vitro*. Whole cell lysate, 30 μg protein loaded per lane. Densitometries for western blots **(A-B)** depict the relative protein expression, normalized to actin and total Akt. Phosphorylated Akt increased significantly in doxorubicin-naïve (dox-naïve), compared to doxorubicin-resistant MCF7 cells (dox-res), at AKTi concentrations above 0.5 μM. *p<0.05. **(C-D)** Western blot analysis of Akt and downstream signaling in doxorubicin-naïve or doxorubicin-resistant MB231 **(C)** and MCF7 **(D)** human breast cancer cells, after 24 hrs exposure to A-443654 (MB231: 1 μM, MCF7 0.5 μM) *in vitro*. Densitometries for western blots **(C-D)** depict the relative protein expression, normalized to actin. Bars represent the mean protein expression in experiments performed in triplicate ± SEM.

First, it was established that the IC30 concentration of A-443654 was 1.0 μM in MB231 and 0.5 μM in the MCF7 cell line (Supplementary Figure 2C). A-443654 is a known ATP competitive inhibitor of Akt, which causes a transient increase in Akt phosphorylation at S473 [15]. In line with this, 2 hrs exposure to A-443654 increased Akt phosphorylation in a dose-dependent manner in MCF7 as well as MB231 cells (Figure 2A-2B). The induction of Akt by A-443654 in ER negative MB231 cells was not influenced by doxorubicin resistance (Figure 2A). However, in ER positive MCF7 cells, the induction of Akt phosphorylation by A-443654 was significantly less prominent in doxorubicin-resistant compared to doxorubicin-naïve cells (Figure 2B), suggesting that long-term doxorubicin exposure exhausts the ability to activate Akt and could influence the response to A-443654. Of notice, the baseline phosphorylation level of Akt in doxorubicin-naïve MB231 differed in Figure 1A and 2A, possibly due to the use of different dissolvents given to control cells in the two experiments; HPMC was used as dissolvent for A-443654 (Figure 2A), whereas DMSO was the dissolvent for doxorubicin (Figure 1A).

To decipher the consequence of Akt inhibition in a wider time frame, Akt phosphorylation and downstream signaling was assessed after 24 hrs of A-443654 (IC30) exposure (Figure 2C-2D). As compared to 2 hrs, total Akt was profoundly reduced after 24 hrs, suggesting the Akt inhibitor may induce protein degradation. In parallel, phosphorylated Akt remained upregulated after 24 hrs of doxorubicin exposure in the doxorubicin-naïve as well as the doxorubicin-resistant MCF7 and MB231 cell lines, which is in accordance with the reported activity of A-443654 [16]. With respect to downstream signaling, it was clearly reduced by A-443654 in the doxorubicin-naïve MCF7 cell line, with decreased GSK3 and S6K phosphorylation and increased 4EBP1 protein levels (Figure 2D), and the same signaling inhibition was observed in the doxorubicin-resistant MCF7 cell line. The influence of A-443654 on Akt downstream signaling was less pronounced in the MB231 cell line (Figure 2C). Whereas reduced GSK3 and increased 4EBP1 phosphorylation was observed in doxorubicin-naïve MB231, Akt inhibition had no influence on phosphorylated S6K. In doxorubicin-resistant MB231, A-443654 reduced S6K and increased 4EBP1 phosphorylation, in accordance with protein synthesis inhibition, but at the same time phosphorylated GSK3 protein levels increased, indicating glycogen synthase and cell cycle activation. All in all, these results point to a stronger dependence on Akt downstream signaling for cell proliferation in MCF7 than in MB231 breast cancer cells when doxorubicin resistance evolves.

**Figure 3:**
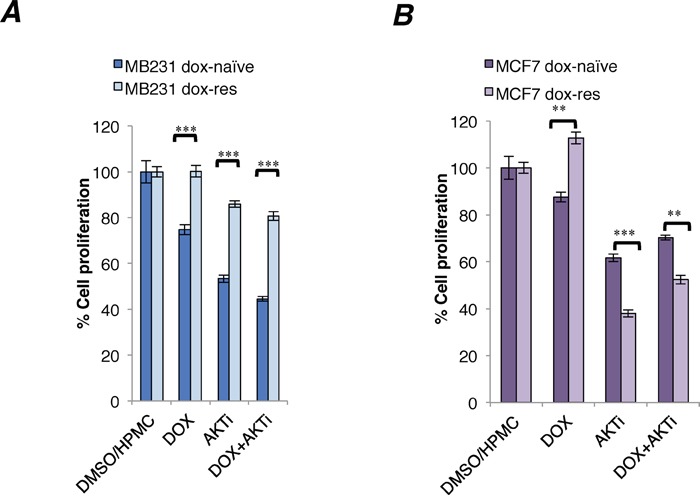
Cytotoxicity of A-443654 and doxorubicin in doxorubicin-naïve and doxorubicin-resistant human breast cancer cell lines *In vitro* cytotoxicity of doxorubicin (1 μM), Akt inhibitor A-443654 at IC30 concentration, or the combination, in doxorubicin-naïve or resistant MB231 **(A)** and MCF7 **(B)** cells, after 24 hrs drug exposure. WST-1 cell proliferation assay, absorbance read at optical density (OD) 450 nm, normalized to readings in control wells exposed to equivalent volumes of DMSO (doxorubicin stock solvent) and HPMC (dissolvent for A-443654). Bars depict the mean ± SEM.**p<0.01, ***p<0.001

Indeed, the Akt inhibitor exhibited significantly increased cytotoxicity in doxorubicin-resistant compared to doxorubicin-naïve MCF7 cells (Figure 3B). In contrast, the cytotoxicity of A-443654 was significantly reduced in doxorubicin-resistant compared to doxorubicin-naïve MB231 cells (Figure 3A). The cytotoxicity of A-443654 was not augmented by doxorubicin in neither cell line (Figure 3A-3B).

### Efficacy of doxorubicin and Akt inhibition in doxorubicin-naïve MB231 and MCF7 xenografts *in vivo*

The efficacy of sham treatment, A-443654 or doxorubicin, alone or in combination, was assessed in NOD/SCID mice implanted orthotopically with doxorubicin-naïve MB231 or MCF7 human breast cancer (n=5-6 mice/group).

**Figure 4:**
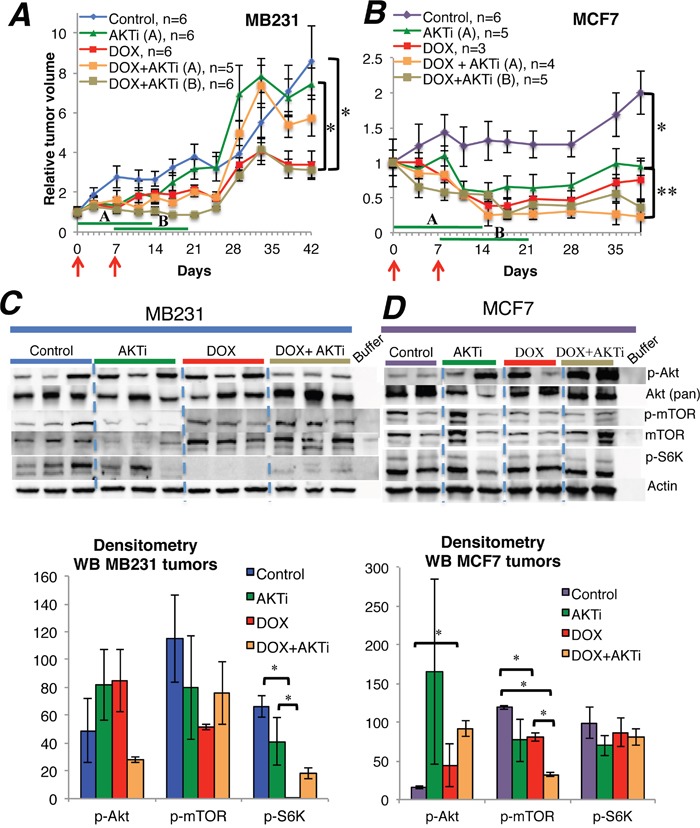
The influence of A-443654 and doxorubicin on tumor growth *in vivo* **(A-B)** Tumor growth of MB231 and MCF7 breast cancer in NOD/SCID mice, given doxorubicin (DOX) 1.25 mg/kg i.p. qW twice (red arrows), Akt inhibitor A-443654 (AKTi) 3.75 mg mg/kg BID 14 days (green lines) or the combination. AKTi treatment commenced either at the first **(A)** or at the second **(B)** doxorubicin injection. Tumor volume is displayed as the mean ± SEM for each group, relative to tumor volume on the day treatment started. *p<0.05, **p<0.01. **(C-D)** Western blots for PTEN and Akt-mTOR-S6K signaling in MB231 **(C)** and MCF7 **(D)** tumors, harvested the last day of A-443654 treatment. Whole cell lysate, 30 μg protein loaded per lane. The sample order on the blot pictures has been rearranged to enhance readability. Densitometries for western blots **(C-D)** depict the relative protein expression, normalized to actin. Phosphorylated Akt (p-Akt) and mTOR (p-mTOR) were normalized to actin and thereafter to total Akt and mTOR, respectively. Bars represent the mean protein expression for experiments performed in duplicate **(D)** or triplicate **(C)** ± SEM. *p<0.05

The Akt inhibitor A-443654 was ineffective as monotherapy in MB231 tumors in mice, but inhibited tumor growth significantly in MCF7 tumors (Figure 4A-4B). Doxorubicin treatment yielded significant tumor inhibition in both cancer subtypes. In MB231, the combination of doxorubicin and A-443654 inhibited tumor growth significantly compared to A-443654 or sham treatment, but only if A-443654 was postponed for a week after commencing doxorubicin administration (treatment group B). In contrast, co-administration of A-443654 and chemotherapy (treatment group A) diminished the tumor growth inhibition induced by doxorubicin alone. In MCF7, the combination of doxorubicin and A-443654 yielded significant tumor growth inhibition as compared to A-443654 or sham treatment, but only if the Akt inhibitor and doxorubicin were administered concomitantly (treatment group A). In both breast cancer models there was no significant difference in tumor response between doxorubicin alone and doxorubicin combined with A-443654.

Subcutaneous Akt inhibitor injections caused a 7% weight loss after 14 days of treatment, which was comparable to combined treatment with doxorubicin and A-443654. However, the observed weight loss precluded further extension of the A-443654 treatment period, to assess whether long-term Akt inhibiton could augment the efficacy of doxorubicin. Unfortunately, two mice in the doxorubicin and one mouse in the doxorubicin and A-443654 group (A) had to be euthanized and taken out of the MCF7 trial due to accidental injection of doxorubicin into the gut wall and subsequent gut necrosis.

In a separate experiment, mice exposed to the same treatment regimens as above were euthanized after 14 days and tumor tissue extracted for molecular analysis (doxorubicin-naïve MB231; n=3 mice/group and MCF7; n=2 mice/group). As monotherapy, A-443654 or doxorubicin yielded a heterogenous increase in Akt phosphorylation in MCF7 and to a lesser extent in MB231 xenografts, although not significant by densitometry (Figure 4C-4D). Combined treatment with A-443654 and doxorubicin increased Akt phosphorylation in MCF7 xenografts significantly, whereas the phosphorylation level of Akt in MB231 was unaffected by the combination regimen (Figure 4C-4D). While Akt phosphorylation increased substantially subsequent to 24 hours of A-443654 treatment *in vitro* (Figure 2A-2D), this increase was less pronounced in MB231 and MCF7 xenografts after two weeks of A-443654 treatment (Figure 4C-4D).

Potential effects of Akt inhibition was further monitored by analyzing downstream target effects (S6K phosphorylation status). In doxorubicin-naïve MB231 tumors, protein analysis demonstrated significantly reduced S6K phosphorylation after treatment with doxorubicin alone or combined with A-443654 (Figure 4C). In doxorubicin-naïve MCF7 tumors, A-443654 or doxorubicin, either alone or in combination, reduced mTOR phosphorylation (Figure 4D). Gene expression analysis of *PTEN, AKT1* and *S6K* in tumors extracted 14 days after commencing therapy (Supplementary Figure 3) demonstrated a significant decrease in *AKT1* in MCF7 tumors subsequent to doxorubicin exposure, but apart from this no definite differences between the treatment groups were observed in neither MCF7 nor MB231.

### Gene expression changes induced by anthracyclines in human breast cancers

**Figure 5:**
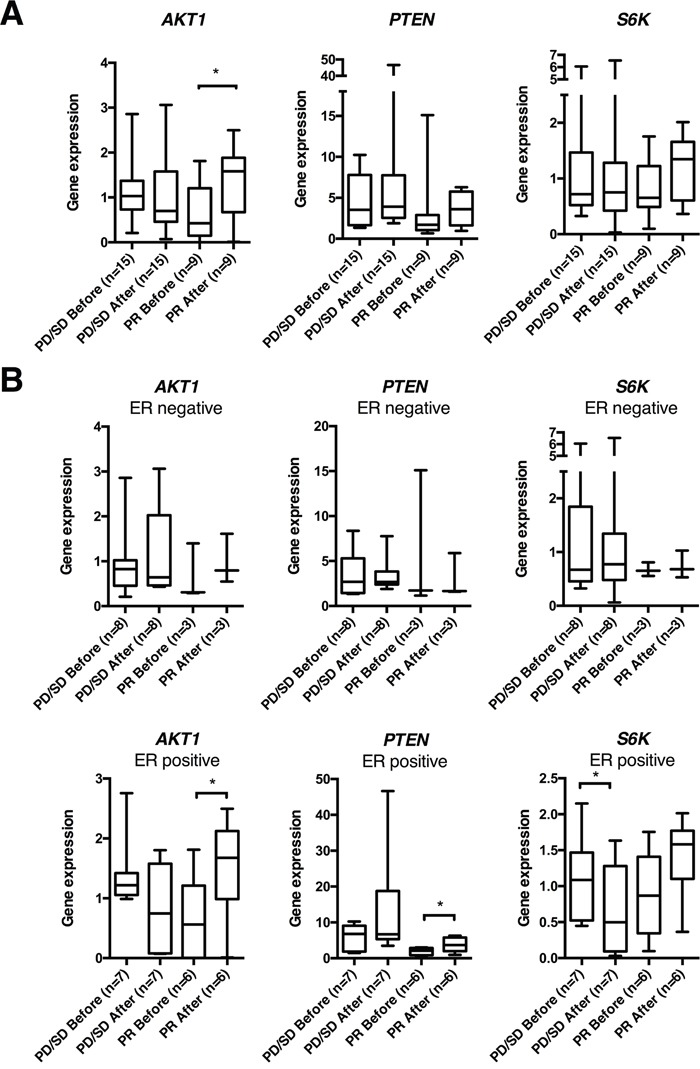
*AKT1*, *PTEN* and *S6K* gene expression in human breast cancers before and 24 hrs after epirubicin exposure **(A)** Box plots of gene expression of *PTEN*, *AKT1* and *S6K* normalized to *RPLP2* in human breast cancer samples, before and 24 hrs after the first epirubicin dose, from patients in the dose dense trial. **(B)** Box plots from the same patient cohort as in **(A)**, but depicted separately for estrogen receptor (ER) positive and ER negative breast cancers. *p<0.05

Next, to compare with the preclinical results, we examined how anthracyclines affected acute and chronic tumor gene expression by analyzing breast cancer samples obtained before and 24 hours after the first epirubicin (60 mg/m^2^ i.v.) course, or before and after 16 weeks of weekly doxorubicin (14 mg/m^2^ i.v.). All 24 tumors collected in the dose dense epirubicin trial (ClinicalTrials.gov NCT00496795) expressed *PTEN, AKT1* and *S6K*, before and/or after treatment (Supplementary Figure 4). Among the patients treated with epirubicin, *AKT1* gene expression increased significantly (p=0.016) in tumors that subsequently regressed on treatment (PR; n=9), whereas no change was observed in tumors that did not respond (SD, PD; n=15, Figure 5A). The mRNA levels of *PTEN* and *S6K* did not change significantly, neither among responders nor non-responders (Figure 5A). Stratifying patients according to ER status, neither *AKT1*, *PTEN* nor *S6K* mRNA levels were influenced by epirubicin exposure among ER negative tumors (n=11), independent of clinical response to therapy (Figure 5B). Interestingly, among the ER positive tumors (n=13), *AKT1* (p=0.040) as well as *PTEN* levels (p=0.039) increased significantly in patients who responded to therapy (n=6); whereas no change in neither *AKT1* nor *PTEN* levels was observed among the non-responders (n=7, Figure 5B). Furthermore, *S6K* decreased significantly (p=0.027) in ER positive tumors that did not respond to therapy (n=7, Figure 5B).

After a median follow-up of 69 months, six out of 24 patients from the dose dense trial had developed breast cancer recurrence; no difference in gene expression changes between patients relapsing and those not relapsing was observed (Supplementary Figure 4).

**Figure 6:**
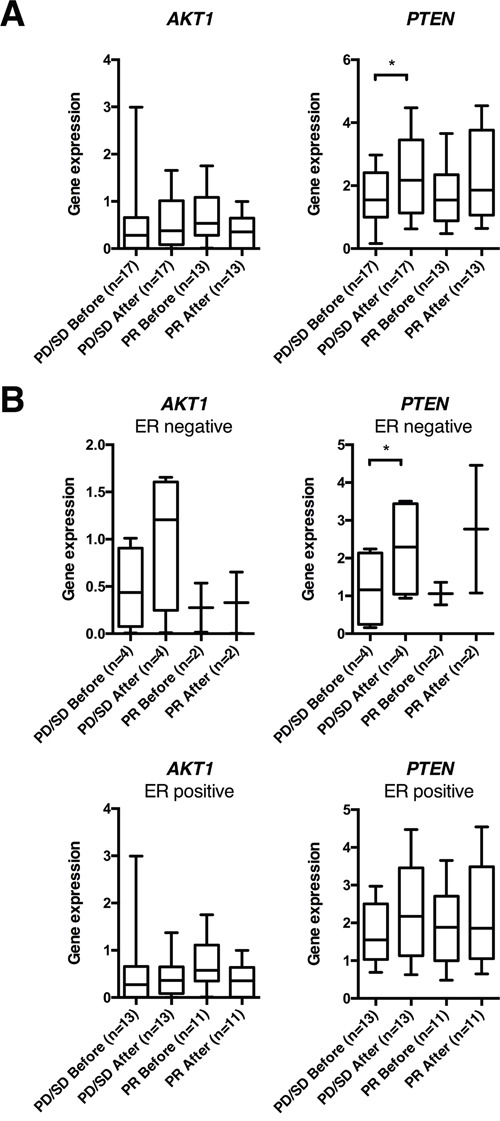
*AKT1* and *PTEN* gene expression in human breast cancers before and after 16 weeks of doxorubicin treatment **(A)** Box plots of gene expression of *PTEN* and *AKT1* normalized to *RPLP2* in human breast cancer samples, before and after 16 weeks of doxorubicin treatment, from patients in the doxorubicin trial. **(B)** Box plots from the same patient cohort as in **(A)**, but depicted separately for estrogen receptor (ER) positive and negative breast cancers. *p<0.05

To assess potential long-term effects of anthracycline treatment, tumor samples collected from 30 patients with locally advanced breast cancers, before and after 16 weeks of doxorubicin [30, 31], were examined for long-term gene expression changes of *PTEN* and *AKT1* (Supplementary Figure 5). Analysing all patients together, no change in neither *AKT1* nor *PTEN* expression was observed. However, stratifying patients based on response to therapy, *PTEN* expression increased significantly (p=0.033) among non-responders (patients having a PD or SD on therapy; n=17), in particular among ER negative non-responders (n=4; p=0.026; Figure 6A-6B). In contrast, while 22 out of 30 patients had relapsed during a median follow-up of 235 months, gene expression changes did not correlate to long-term outcome (Supplementary Figure 5).

The mutation status of *PIK3CA* and *TP53* was assessed in all the above tumor samples (Supplementary Figure 4 and Supplementary Figure 5). The low number of tumors harboring *PIK3CA* mutations precluded any correlation analysis between *PIK3CA* mutation status and the gene expression changes observed. Among tumors assessed before and 24 hours after the initial epirubicin course, no difference in *PTEN* or *AKT1* gene expression was observed regardless of response to chemotherapy, if subdivided into *TP53* wt (n=16) and *TP53* mutated tumors (n=8). Upregulated *AKT1* 24 hrs after the first epirubicin exposure was observed in six out of 13 ER positive breast cancers which subsequently regressed on this treatment (Figure 5); and four out of these six tumors where *AKT1* increased harbored wt *TP53* status (Supplementary Figure 4).

In tumors examined before and after 16 wks of doxorubicin, if split by *TP53* mutation status (*TP53* wt; n=15, *TP53* mutated; n=15), *PTEN* increased significantly after treatment (p=0.02) in tumors harboring *TP53* mutations that did not respond to doxorubicin treatment (n=8), whereas no change was observed among responders (n=7) or among *TP53* wt tumors (data not shown). Also, there was no significant change in *AKT1*, among responders or non-responders to doxorubicin, if the subgroups were split by *TP53* mutation status.

## DISCUSSION

Patients with ER positive as well as ER negative breast cancer obtain improved survival from adjuvant polychemotherapy [17], but the benefit of chemotherapy is less in typical luminal A tumors with strong ER expression compared to other subtypes [18]. The mutational landscape of breast cancer subtypes differ substantially, with a high prevalence of activating *PIK3CA* mutations in ER positive, luminal or HER2-enriched tumors, whereas inactivating *TP53* mutations are commonly observed in ER negative subtypes [4]. Moreover, while activating *AKT1* mutations are rare in human breast cancers, they occur more frequently among luminal or HER2-enriched (2-4%) than basal-like tumors (0%) [4], suggesting a selection pressure towards increased Akt signaling in these neoplasms. Accordingly, the PI3K-Akt-mTOR pathway has been targeted therapeutically to counteract acquired resistance to endocrine therapy and combined trastuzumab-chemotherapy in clinical trials [1, 3, 5, 19]. However, the importance of PI3K-Akt-mTOR signaling to chemoresistance has not been fully elucidated.

Here, we systematically explored alterations in this pathway in response to anthracycline and/or Akt inhibition in ER positive and negative breast cancer cell lines and their concomitant xenografts and to anthracycline treatment in human breast cancers.

We established that the Akt inhibitor A-443654 reduces cell proliferation both in the ER positive MCF7 and the ER negative MB231 cell line *in vitro*. However, Akt inhibition yielded tumor regression in MCF7 and not MB231 *in vivo*, and doxorubicin significantly augmented this tumor response only in MCF7 xenografts. Of notice, A-443654 caused significant weight loss which was intolerable beyond 14 days, and we therefore could not explore its full potential alone or combined with anthracyclines. Toxicity was a similar problem in the first clinical trials testing Akt inhibitors, although next generation compounds seem better tolerated [20] and should be tested in long-term combination schedules with anthracyclines. Importantly, the combined efficacy of A-443654 and doxorubicin was dependent on timing, where concomitant administration of the Akt inhibitor and chemotherapy was required for optimal tumor regression in MCF7 xenografts. In contrast, co-administration of A-443654 with doxorubicin reduced the efficacy of doxorubicin in MB231 xenografts. Doxorubicin yielded rapid upregulation of phosphorylated Akt in MCF7 cells *in vitro*, whereas long-term exposure and induction of doxorubicin resistance was required to upregulate phosphorylated Akt in MB231. This may explain the different efficacy with respect to timing between doxorubicin and the Akt inhibitor in the two cell lines.

Previous studies have revealed A-443654 to act as an ATP competitive inhibitor of Akt; as such, it increases Akt phosphorylation while at the same time inhibiting Akt downstream signaling [15, 16]. Accordingly, A-443654 rapidly increased Akt phosphorylation, which was more pronounced in the MCF7 as compared to the MB231 cell line, and suggesting a particular responsiveness of the PI3K-Akt-mTOR pathway in ER positive breast cancer cells. However, the cytotoxicity of A-443654 was comparable between MCF7 and MB231, and similar inhibition of Akt downstream signaling was observed in the ER positive and ER negative cell lines. In contrast, the activity of A-443654 was profoundly reduced in doxorubicin-resistant MB231 cells, where the inhibition of Akt signaling by A-443654 was abrogated. Interestingly, doxorubicin resistance enhanced the cytotoxicity of A-443654 significantly in MCF7, with maintained inhibition of Akt downstream signaling. The mechanisms behind the enhanced activity of A-443654 in doxorubicin-resistant MCF7 cells remain to be established. However, the baseline Akt phosphorylation level is higher in the doxorubicin-resistant MCF7 cell line, as compared to doxorubicin-naïve MCF7 cells, potentially explaining the increased cytotoxicity towards A-443654.

In line with previous observations [10, 21], we established that doxorubicin exposure increases Akt phosphorylation in the ER positive MCF7 and T47D human breast cancer cell lines, but not the ER negative MB231 cell line. Herein we expand upon these data to show that MCF7 cells made resistant to doxorubicin exhibit a higher constitutive Akt phosphorylation levels which is not affected by further doxorubicin exposure. The increased cytotoxicity of Akt inhibition in doxorubicin-resistant MCF7 breast cancer cells points to a potential use of such a drug class in ER positive breast cancer, in particular if resistance to anthracycline has developed and Akt phosphorylation levels are elevated. Importantly, while the Akt inhibitor exhibited increased cytotoxicity in doxorubicin-resistant compared to doxorubicin-naïve MCF7 breast cancer, we did not examine whether Akt inhibitors can be used to reverse doxorubicin resistance, but this issue should be addressed in future trials. However, upregulated PI3K-Akt-mTOR signaling is clearly associated with chemoresistance, which has been shown in various preclinical cancer models, and chemotherapy response can be augmented in this setting by simultaneous PI3K or Akt inhibition [10, 11, 22–24], in particular in ER positive breast cancer [10, 22].

Finally, we provide clinical data demonstrating that increased *AKT1* gene expression 24 hours after epirubicin exposure characterizes ER positive, but not ER negative, primary breast cancers that subsequently regress on anthracycline treatment. Interestingly, no change in tumor *AKT1* expression was observed in patients after 16 weeks of doxorubicin, suggesting an intermittent Akt response where the efficacy of Akt inhibitors could depend on timing. Furthermore, an increased *AKT1* mRNA level 24 hrs after the first chemotherapy course could potentially be used as a biomarker identifying ER positive tumors likely to respond to chemotherapy. The reason why elevated *AKT1* mRNA is associated with good response remains to be elucidated. However, if high *AKT1* translates into increased Akt activation throughout the duration of chemotherapy, chronic Akt activation may promote senescence and apoptosis by downregulating MDM2 and increasing p53 in breast cancers with preserved p53 function [25, 26]. Of notice, among the ER positive breast cancers which responded to epirubicin, four out of six tumors with upregulated *AKT1* after chemotherapy harbored wt *TP53* status.

In contrast to the patient data, *AKT1* mRNA levels were not affected in the MB231 and MCF7 human breast cancer cell lines after 24 hrs chemotherapy exposure. The reason for this discrepancy remains to be elucidated, but could be due to the admixture of tumor cells and stroma in patient tumor samples in contrast to the pure tumor cell content in the *in vitro* cultures. Unfortunately, we did not have patient samples available for proteinanalysis of Akt and Akt signaling to compare with the gene expression data. Furthermore, the induction of Akt phosphorylation by A-443654 which was observed in MB231 and MCF7 *in vitro*, was not detected in the corresponding xenografts. Again, the admixture of tumor cells and stroma cells *in vivo*, as well as the heterogeneity between the xenografts may explain the lack of correlation between the *in vivo* and *in vitro* findings. Moreover, the xenografts used for the proteinanalysis were extracted after 14 days of treatment compared to the 2 hrs and 24 hrs of treatment in the *in vitro* experiments. The reason why decreased *PTEN* mRNA levels did not decrease PTEN protein levels in MB231 cells after 24 hrs exposure to doxorubicin also remains to be established. Whereas rapid changes in gene expression are induced by the chemotherapy, protein changes may take longer to develop due to the relatively long half-life of PTEN (>8 hrs) [13]. Furthermore, there is no strong correlation between *PTEN* mRNA and PTEN protein levels in human breast cancer [14], which could be explained by post-transcriptional and post-translational mechanisms modifying protein expression and stability.

In conclusion, our data point to upregulated Akt expression as a recurrent initial response to anthracyclines in ER positive human breast cancers, and in particular, among patients who respond to chemotherapy. Furthermore, we observed increased sensitivity to Akt inhibition in doxorubicin-resistant, compared to doxorubicin-naïve, ER positive MCF7 breast cancer cells. Accordingly, the benefit of Akt inhibition is clearly context-dependent, with respect to ER status and previous anthracycline exposure. Thus far, the role of Akt inhibitors to augment the efficacy of chemotherapy in solid tumors has not been dealt with to a large extent, despite promising preclinical and clinical data [23, 24, 27–29] and should be explored further, in particular in ER positive breast cancers.

## MATERIALS AND METHODS

### Ethical declaration

The authors declare that the experiments within this paper comply with the ethical standards and current laws in Norway.

### Cell lines

The ER positive MCF7 and T47D and the ER negative MDA-MB-231 (MB231) human breast cancer cell lines were used for all the preclinical experiments. For cell growth conditions and cell line identity, see *Supplementary methods*. Doxorubicin (Adriamycin, Pfizer) was diluted to 2 mg/ml in DMSO 99% for cell culture experiments, stored as frozen aliquots (-20°C), and prepared fresh by dilution in complete cell culture medium for each experiment to preserve drug stability. Control cells were always incubated with an equivalent volume of DMSO 99% as cells exposed to medium containing doxorubicin. A-443654 (AbbVie) was dissolved in 0.2% hydroxypropyl methylcellulose (HPMC, Sigma) prior to use. For comparison, control cells were given an equivalent volume of HPMC as those cells exposed to A-443654.

### Generation of doxorubicin-resistant cell lines

Doxorubicin-naïve MB231 and MCF7 cells were grown in gradually increasing doxorubicin concentrations over several months. When the cells were subconfluent they were exposed to growth medium containing twice the previous concentration of doxorubicin, and this was repeated until a dose was reached, where the cells would not expand any further. At this point, the cells had acquired resistance to doxorubicin 1.5 μM (MB231 dox-res) and doxorubicin 0.65 μM (MCF7 dox-res), each by exposure for 48 hrs. The cells were maintained in doxorubicin-free medium, but exposed to doxorubicin at their resistance dose every two weeks to maintain resistance. Control cells were propagated in medium with an equivalent volume of DMSO. To determine the acute response to doxorubicin in doxorubicin-resistant cells, the cells were seeded at 3×10^5^ in 6-well dishes (Nunc) and incubated for 24 hrs until 70% confluence. The medium was then replaced by medium containing either doxorubicin or DMSO, and cells were incubated for another 24 hrs before the cells was harvested and RNA and protein isolated.

### *In vitro* activity and cytotoxicity of A-443654 and doxorubicin

Subconfluent MB231 and MCF7 cells (in T25 flasks), either doxorubicin-naïve or doxorubicin-resistant, were exposed to increasing concentrations of Akt inhibitor A-443654 or HPMC (control) to assess the influence on Akt phosphorylation, and protein was harvested after 2 hrs. To evaluate the influence of the Akt inhibitor on Akt phosphorylation and downstream signaling after 24 hrs, cells were exposed to A-443654 at the IC30 (1 μM MB231, 0,5 μM MCF7), before harvesting protein.

To assess the influence of doxorubicin +/- Akt inhibitor A-443654 on cell viability, 5000 MB231, 1500 MCF7 or 20000 T47D cells were seeded per well in 96-well plates (Falcon), and allowed to attach over night in complete medium. Thereafter the drug(s) was added, before culturing the cells for another 24 hours. Cell proliferation after drug exposure was assessed by the WST-1 assay (Roche), as described in the manufacturer´s manual.

### Gene expression analysis and western blots

Therapy-induced changes in gene and protein expression were assessed using qPCR and western blot analyses. Detailed methods, primers and antibodies are described in *Supplementary methods*.

### *In vivo* cytotoxicity of A-443654 and doxorubicin

NOD/SCID mice were bred and mouse crossings performed inhouse at the Animal Facility, University of Bergen. Adult mice of fertile age were anesthetized with isoflurane (Baxter) before 1×10^6^ MCF7 or MB231 cells were injected orthotopically in the fourth left inguinal mammary gland. The tumors were measured every 3-4 days using Vernier calipers, and tumor volumes were calculated using the formula a^2^b/2, where a and b are the shorter and longer diameter of the tumors respectively. Treatment commenced when the tumors had reached 4-6 mm in diameter, and the animals were stratified into groups according to tumor size. The mice were euthanized if signs of serious distress occurred or when the first tumor in any treatment group had a tumor diameter exceeding 17 mm.

The maximum tolerable dose (MTD) of doxorubicin, given weekly for two following weeks, with or without the Akt inhibitor A-443654 was initially assessed in non-tumor bearing NOD/SCID mice before commencing the therapy trial in xenograft-implanted mice. Doxorubicin was dissolved in 0.9% NaCl (Baxter) and injected i.p. once weekly for two consecutive weeks to establish an MTD of 1.25 mg/kg qW. A-443654 was dissolved in HPMC and administered subcutaneously at 3.75 mg/kg BID for 14 consecutive days, based on dosage reported elsewhere [16]. In the combination treatment groups, administration of A-443654 commenced either upfront (A), 24 hours after the first doxorubicin injection (to treat primary resistance due to high Akt signaling), or started as a delayed regimen (B) at the same time as the second doxorubicin injection (to counteract doxorubicin-induced acquired resistance due to upregulated Akt signaling). Control mice were given 0.2% HPMC s.c. and 0.9% NaCl i.p.

In a separate experiment, three MB231 mice and two MCF7 mice per group were sacrificed at the end of 14 days of treatment (as given in the treatment trial above) for protein analysis. All animals were euthanized by cervical dislocation two hours after the last injection of A-443654 or sham treatment, and tissue samples from the tumor as well as all organs snap-frozen on liquid N_2_ and stored at -80°C.

The animal experiments were performed with the approval of and in accordance with guidelines by the Norwegian State Commission for Laboratory Animals.

### Patient breast cancer samples

The short-term effect of anthracyclines on *PTEN*, *AKT1* and *S6K* gene expression was assessed in a selected cohort of 14 ER positive and 11 ER negative breast cancers biopsied before and 24 hrs after receiving their first course of epirubicin as part of the “dose dense trial” (ClinicalTrials.gov NCT00496795) wherein treatment-naïve patients with locally advanced breast cancer were given dose dense neoadjuvant epirubicin 60 mg/m^2^ q2w (four courses) followed by docetaxel 100 mg/m^2^ q2w (four courses). The presence of pre-treatment *PIK3CA* and *TP53* mutations in all tumor samples were examined as described in *Supplementary methods*. One ER positive tumor pair was excluded due to poor RNA quality, leaving 24 tumor pairs for analysis.

Furthermore, to analyze for long-term effects of anthracyclines on *PTEN* and *AKT1* mRNA levels, gene expression was assessed in paired tumor samples from 30 patients with treatment-naïve locally advanced breast cancer in the “doxorubicin trial”, biopsied before and after 16 wks of neoadjuvant doxorubicin 14 mg/m^2^ qW treatment [30, 31]. Briefly, tumor pairs for mRNA analysis were available from 24 patients with ER positive and six patients with ER negative locally advanced breast cancer.

The dose dense and doxorubicin clinical trials were approved by the Regional Ethical Committee of the Western health region in Norway (reference numbers: 192/91-69.91 and 079.06). All patients gave their informed consent before inclusion. Accordingly, all procedures performed in these clinical trials were in accordance with the national ethical standards and with the 1964 Helsinki declaration and its later amendments.

### Statistics

SPSS 22/PASW 17.0 and Graph Pad Prism v6 software packages were used for statistical analyses. Correlation analysis between *AKT1* and *PTEN* or *S6K* mRNA expression levels was performed using Spearman´s rho. Comparison of gene or protein expression levels was performed using the Student's *t*-test for paired samples or two independent samples, as appropriate. All p-values reported are two-tailed, and p<0.05 was considered statistically significant.

### Availability of data and materials

All raw data generated from the experiments presented are available from the corresponding author upon request. The data subset used for mutational calling of *TP53* and *PIK3CA* in the “dose dense trial” was extracted from whole exome sequencing data, and the DNA sequences are available from the corresponding author upon request.

## SUPPLEMENTARY MATERIALS FIGURES AND TABLES



## Abbreviations

4EBP1: 4E-binding protein 1
AKTi: Akt inhibitor
ATCC: American Type Culture Collection
ER: Estrogen Receptor
Dox-res: Doxorubicin resistant
HPMC: Hydroxypropyl Methylcellulose
MB231: MDA-MB-231
mTOR: mammalian Target Of Rapamycin
MTD: Maximum Tolerable Dose
p-Akt: phosphorylated Akt at Ser473
PBS: Phosphate-Buffered Saline
p-GSK3α/ß: phosphorylated glycogen synthase kinase 3α/ß at Ser21/9
PI3K: Phosphatidylinositol-4,5-bisphosphate 3-kinase
p-mTOR: phosphorylated mammalian target of rapamycin at Ser2448
PTEN: phosphatase and tensin homolog
p-S6K: phosphorylated S6K at Ser371
RPMI: Roswell Park Memorial Institute Medium-1640
RT-PCR: Reverse Transcriptase Polymerase Chain Reaction
S6K: S6 Kinase
STR: Short Tandem Repeat
TP53: Tumor Protein p53
qPCR: Quantitative PCR
