# Supplementary material for: Activation of Akt characterizes estrogen receptor positive human breast cancers which respond to anthracyclines
Source: Oncotarget. 2017 Apr 17;8(25):41227–41. doi: 10.18632/oncotarget.17167 (PMC5522318; doi:10.18632/oncotarget.17167)
Supplement: Supplementary file 1 [file oncotarget-08-41227-s001.pdf]

# Activation of Akt characterizes estrogen receptor positive human breast cancers which respond to anthracyclines

## SUPPLEMENTARY MATERIALS

## SUPPLEMENTARY METHODS

### Cell lines and growth conditions

The human breast cancer cell lines MDA-MB-231 (MB231), MCF7 and T47D were purchased from American Type Culture Collection (ATCC, Manassas, VA). The cells were grown at 37°C in 5% $CO_2$ atmosphere in Roswell Park Memorial Institute medium-1640 (RPMI), supplemented with non-essential amino acids, 10% fetal calf serum, 100 U/ml penicillin, 100 µg/ml of streptomycin and 400 uM L-glutamine (all products: Lonza, Basel, Switzerland). Additionally, the RPMI medium for T47D was supplemented with human insulin 108 U/ml (Life/Thermo Fisher). The cell lines were tested negative for mycoplasma using the Mycoplasma Detection Kit Venor GeM (Minerva Biolabs) before starting the experiments listed. Also, the cell lines were fingerprinted based on short tandem repeat (STR) markers with the AmpFLSTR® Profiler Plus® and AmpFLSTR® COfiler® PCR Amplification Kit, followed by capillary electrophoresis on the ABI PRISM® 310 Genetic Analyzer and STR allele identification using the Peak Scanner Software v1.0 (Applied Biosystems).

### Basic genomic procedures

Breast cancer cells were grown on 6-well plates (Nunc), under conditions as described above, until subconfluency. After exposure to doxorubicin, the cells were rinsed gently with warm (37°C) phosphate-buffered saline (PBS), and RNA was isolated using the Qiagen RNeasy kit, as described in the product manual, followed by DNAse (Ambion) treatment.

The human breast cancer biopsies were snap-frozen immediately after extraction and stored on liquid nitrogen until analysis, while collecting prospectively response rates and patient survival data. DNA for mutation analysis was extracted by the Qiagen DNeasy kit, and RNA was extracted using either Trizol (Invitrogen) (doxorubicin study) or mirVana™ miRNA Isolation Kit, with phenol (Ambion) (dose dense study), followed by DNAse treatment.

RNA concentrations were measured by a NanoDrop spectrophotometer (Thermo Scientific), and cDNA was made from 500 ng of RNA using qScript reverse transcriptase and the qScript cDNA SuperMix with a blend of random and oligo(dT) primers (QuantaBio).

RT-PCR was undertaken using AmpliTaq Gold polymerase (Invitrogen), with reagents as recommended by the company, and PCR reactions were run with 30-35 cycles and the appropriate temperature settings.

Real-time qPCR was performed on a Light Cycler 480 (Roche), with the gene of interest given as the ratio to housekeeping gene RPLP2, and as a mean of three individual runs. Taqman probes were used to detect the PCR products for *PTEN* and *RPLP2*, whereas PowerSYBR®Green (Applied Biosystems) was used for *AKT1* and *S6K*. Lack of gene expression was defined as lack of gene amplification after 35 cycles. Gene of interest was given as a ratio to the housekeeping gene RPLP2, and as a mean of three independent runs, normalized to a cDNA pool of 6 breast cancer cell lines.

All PCR products were checked for specificity by Sanger sequencing.

### Primers for RT-PCR and real-time qPCR

*RT-PCR:*
*AKT1:* F: TTGGCTGCACAAACGAGGGGAGTAC, R: TGCGTTCGATGACAGTGGTCCAC
*β-actin:* F: AGATGACCCAGATCATGTTTG, R: AGGAGCAATGATCTTGATCTTCATTGTG
*PTEN:* F: TTTCCATCCTGCAGAAGAAGC, R: TAAATATGCACATATCATTAC
*S6K:* F: TGGACCATATGAACTTGGCATG, R: CTTTCCATAGCCCCCTTTACC
*Real-time qPCR (PowerSYBR®Green):*
*AKT1:* F: TTGGCTGCACAAACGAGGGGAGTAC, R: TGCGTTCGATGACAGTGGTCCAC
*S6K:* F: TGGACCATATGAACTTGGCATG, R: CTTTCCATAGCCCCCTTTACC
*Real-time qPCR (Taqman):*
*PTEN:*
F: CTTCTCCATCTCCTGTGTAATCAA
R: GTTGACTGATGTAGGTACTAACAGCAT
FAM: CCAGTGCTAAAATTCA
*RPLP2:*
F: GACCGGCTCAACAAGGTTAT
R: CCCCACCAGCAGGTACAC
FAM: AGCTGAATGGAAAAAACATTGAAGACGTC

### *PIK3CA* and *TP53* mutation analysis

*PIK3CA* and *TP53* mutation analysis was performed on pre-treatment breast cancer samples. All tumor samples

in the doxorubicin trial and eight out of 25 tumors in the dose dense trial were analyzed by Sanger sequencing. Briefly, tumor DNA was used as PCR template to assess hot-spot *PIK3CA* mutations in exons 10 and 21 (previously exon 9 and 20) [1] and *TP53* mutations (all exons), using primers as described previously [2, 3]. PCR products were analyzed at Center for Medical Genetics and Molecular Medicine, Haukeland University Hospital, by Sanger sequencing using the BigDye v1.1 reaction mix (Applied Biosystems). The remaining 17 tumors in the dose dense trial were analyzed for *PIK3CA* and *TP53* mutations by massive parallel whole exome sequencing on an Illumina HiSeq 2500, to an average sequencing depth of >200x. Somatic mutations were called using the intersect between the MuTect and Strelka mutation calling algorithms.

### Western blots

Cells and tissues were homogenized and proteins lysed in a custom made total protein lysis buffer (50 mM Tris HCl, pH 7.5, 150 mM NaCl, 0.1% SDS, 1% deoxycholate, 1% Triton X-100) containing a protease inhibitor cocktail (Roche). Protein concentrations were measured by a bicinchoninic acid (BCA) assay (Pierce), and 30 µg protein was loaded per lane for all the immunoblots.

The protein lysates were fractionated using reducing SDS-PAGE gel electrophoresis with Mini-PROTEAN TGX Precast Gels (Bio-Rad), and transblotted by semi-dry technique to nitrocellulose membranes using the Transblot Turbo system (Bio-Rad). Thereafter the membranes were blocked with 5% bovine serum albumine (Sigma) for 60 min, before immunoblotting with the primary antibody. The immobilized antibody was detected using the appropriate horseradish peroxidase-conjugated secondary antibody (Promega) and a 1:5 mix of SuperSignal West Femto and Pico chemiluminescent solution (Pierce). The immunoreaction was visualized on a Fujifilm LAS 4000. Immunoblots for actin were made for all samples to assure equal protein loading for total protein analysis.

Densitometry of western blots was performed using Gel Analyzer 2010a and the band intensity for each protein was normalized to actin. Thereafter phosphorylated Akt and mTOR were normalized to total Akt and mTOR, respectively.

### Antibodies for proteinanalysis

Rabbit anti-actin (Sigma), anti-Akt (pan), anti-phosphorylated Akt (Ser473), anti-mTOR, anti-phosphorylated mTOR (Ser2448), anti-phosphorylated p70 S6K (Ser371), anti-phosphorylated GSK3α/β (Ser21/9) or anti-PTEN and goat anti-4EBP1 (R&D). Actin, 4EBP1 and phosphorylated p70 S6K are polyclonal antibodies, all other antibodies are monoclonal. All primary antibodies were purchased from Cell Signaling, unless noted otherwise.

## REFERENCES

1. Samuels Y, Wang Z, Bardelli A, Silliman N, Ptak J, Szabo S, Yan H, Gazdar A, Powell SM, Riggins GJ, Willson JK, Markowitz S, Kinzler KW, et al. High frequency of mutations of the PIK3CA gene in human cancers. Science. 2004; 304:554.
2. Chrisanthar R, Knappskog S, Løkkevik E, Anker G, Østenstad B, Lundgren S, Berge EO, Risberg T, Mjaaland I, Maehle L, Engebretsen LF, Lillehaug JR, Lønning PE. CHEK2 mutations affecting kinase activity together with mutations in TP53 indicate a functional pathway associated with resistance to epirubicin in primary breast cancer. PLoS One. 2008; 3:e3062.
3. Løes IM, Immervoll H, Sorbye H, Angelsen JH, Horn A, Knappskog S, Lønning PE. Impact of KRAS, BRAF, PIK3CA, TP53 status and intraindividual mutation heterogeneity on outcome after liver resection for colorectal cancer metastases. Int J Cancer. 2016; 139:647-656.

## SUPPLEMENTARY FIGURES

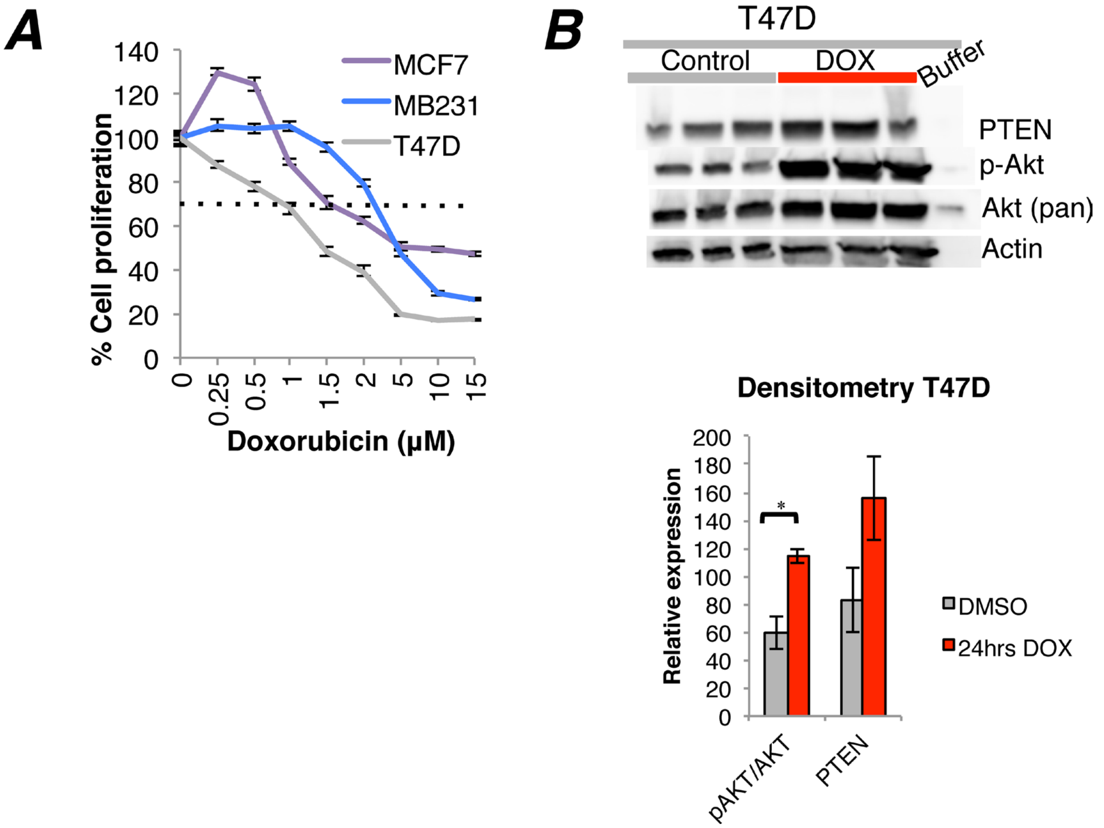


**Supplementary Figure 1: (A)** Doxorubicin cytotoxicity in MB231, MCF7 and T47D breast cancer cells, assessed by WST-1 cell proliferation assay, after 24 hrs drug exposure. Absorbance was read at optical density (OD) 450 nm, and readings normalized to control wells, incubated with an equivalent DMSO (doxorubicin stock solvent) dose as in the wells with the highest doxorubicin dose. Bars depict the mean ± SEM. The dotted line indicates the IC30 level. **(B)** Western blots of PTEN and Akt protein expression in T47D breast cancer cells *in vitro*, after exposure to doxorubicin or DMSO. Drug exposure lasted 24 hrs, at 0.7 µM doxorubicin or an equivalent volume of DMSO (stock solvent for doxorubicin) for control wells, three independent experiments per group. Whole cell lysate, 30 µg protein loaded per lane.

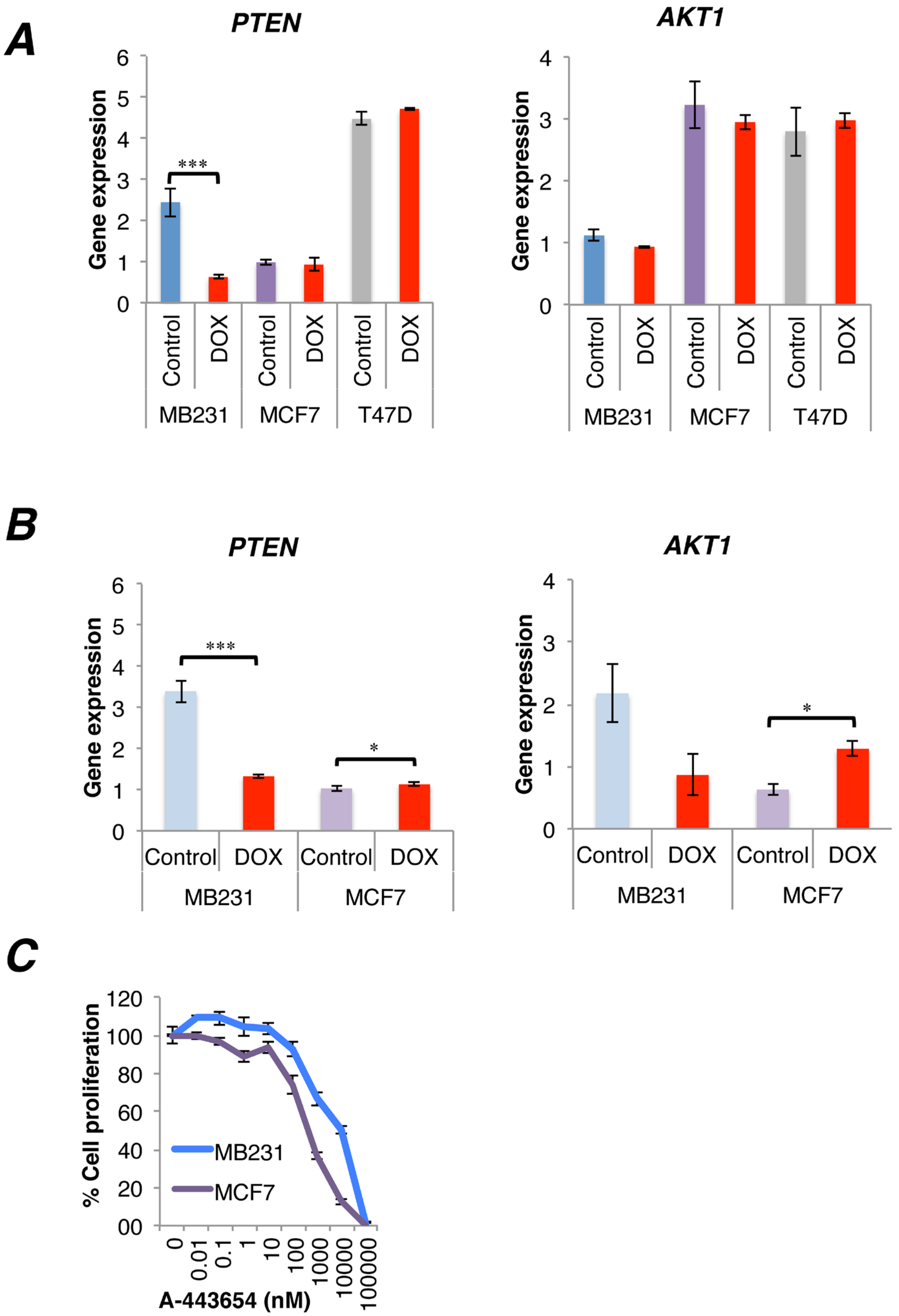


**Supplementary Figure 2: (A)** Gene expression in doxorubicin-naïve MB231, MCF7 and T47D breast cancer cells *in vitro* exposed to doxorubicin (MB231: 1.5 µM, MCF7: 2 µM, T47D: 0.7 µM), 24 hrs drug exposure. **(B)** Gene expression in doxorubicin-resistant MB231 and MCF7 breast cancer cells *in vitro* exposed to doxorubicin (MB231: 1.5 µM, MCF7: 2 µM), 24 hrs drug exposure. Bars depict the mean gene expression of three parallels ± SEM, normalized to *RPLP2,* and analyzed by real-time quantitative RT-PCR. *$p<0.05$. ***$p<0.001$. **(C)** Cytotoxicity of Akt inhibitor A-443654 in MB231 and MCF7 cells, assessed by WST-1 assay, 24 hrs drug exposure. Absorbance was read at optical density (OD) 450 nm, and readings normalized to control wells, incubated with an equivalent dose of HPMC (A-443654 dissolvent) as in the wells with the highest A-443654 dose. Data points depict the mean ± SEM.

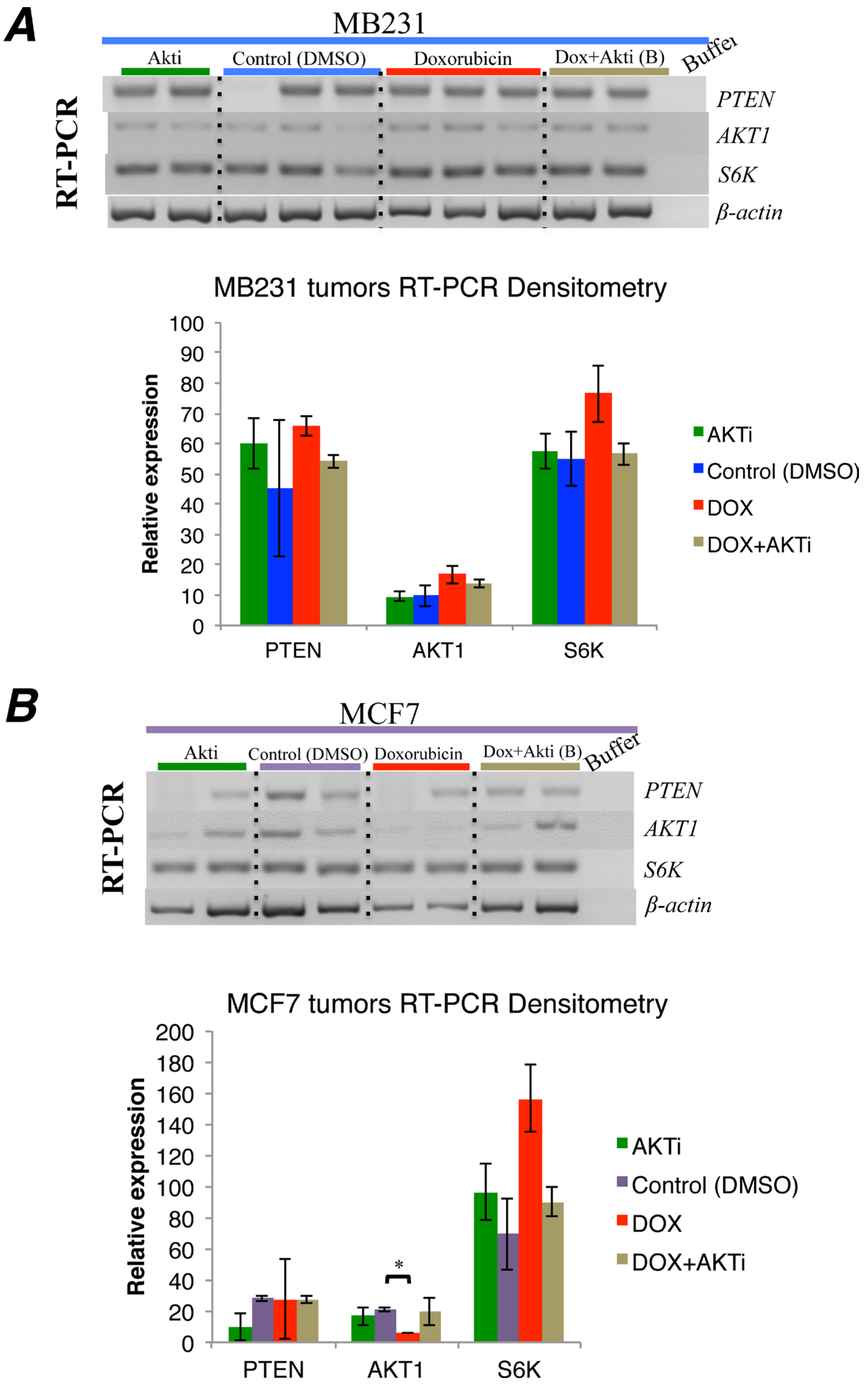


**Supplementary Figure 3:** Gene expression in MB231 **(A)** or MCF7 tumors **(B)** in NOD/SCID mice, given sham treatment (DMSO and HPMC; Controls), doxorubicin (DOX) 1.25 mg/kg i.p. qW twice, Akt inhibitor A-443654 (AKTi) 3.75 mg mg/kg BID 14 days or the combination. Tumors were removed and analyzed 14 days after commencing the treatment. Only tumors from treatment group B, wherein AKTi treatment commenced at the second doxorubicin injection, were used in the current analysis. Densitometry depicts the relative gene expression, normalized to ß-actin. Bars depict the mean ± SEM. *p<0.05.

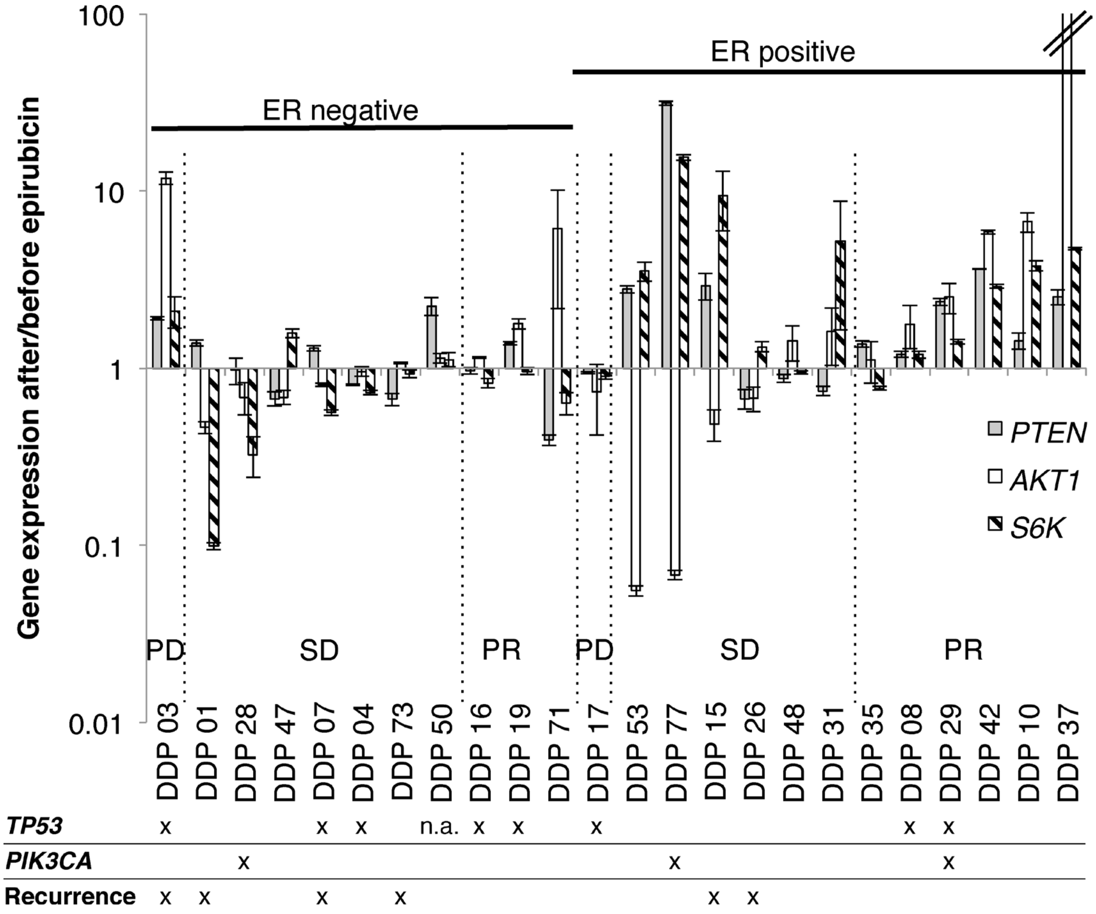


**Supplementary Figure 4: Gene expression in human breast cancer samples before and 24 hrs after the first epirubicin dose (60 mg/m²), in 11 patients with estrogen receptor (ER) negative breast cancer and 13 patients with ER positive breast cancer included in the dose dense trial.** Bars depict the ratio of gene expression after vs. before epirubicin, based on the mean of three separate real-time RT-PCR runs, normalized to *RPLP2* and corrected for cDNA pool. PD: Progressive disease after 4 courses epirubicin q2w, SD: Stable disease, PR: Partial response. Patients with breast cancers harboring *TP53* or *PIK3CA* mutations, and those with breast cancer recurrence are labelled beneath the diagram.

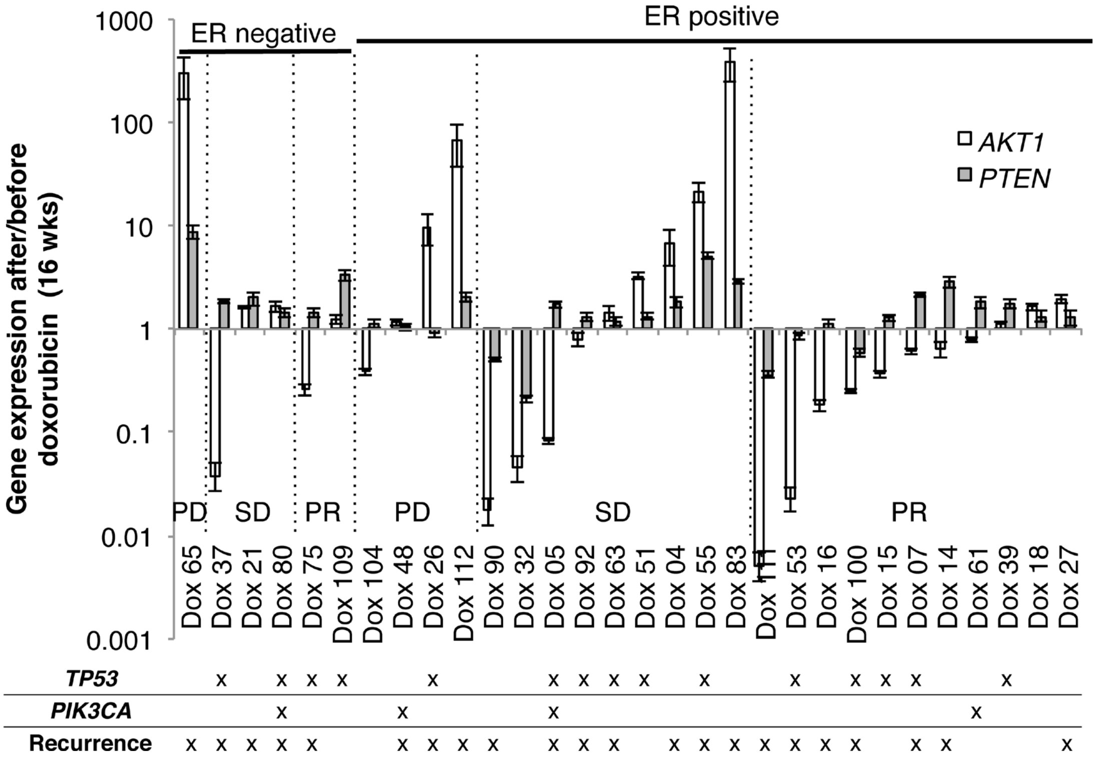


**Supplementary Figure 5: Gene expression in human breast cancer samples before and after 16 weeks of doxorubicin qW 14 mg/m², in six patients with estrogen receptor (ER) negative breast cancer and 24 patients with ER positive breast cancer included in the doxorubicin trial.** Bars depict the ratio of gene expression after vs. before 16 weeks of doxorubicin, based on the mean of three separate real-time RT-PCR runs, normalized to *RPLP2* and corrected for cDNA pool. PD: Progressive disease, SD: Stable disease, PR: Partial response. Patients with breast cancers harboring *TP53* or *PIK3CA* mutations, and those with breast cancer recurrence are labelled beneath the diagram.
